# Effect of pre-season and in-season training on anthropometric variables, somatotype, body composition and body proportion in elite basketball players

**DOI:** 10.1038/s41598-024-58222-4

**Published:** 2024-03-30

**Authors:** A. S. Díaz-Martínez, R. Vaquero-Cristóbal, M. Albaladejo-Saura, F. Esparza-Ros

**Affiliations:** 1 International Chair of Kinanthropometry, UCAM Universidad Católica de Murcia, Murcia, Spain; 2 Department of Physical Activity and Sport Sciences, Faculty of Sport Sciences, University of Murcia, 30720 San Javier, Spain; 3 Facultad de Deporte, UCAM Universidad Católica de Murcia, 30107 Murcia, Spain

**Keywords:** Kinanthropometric, Body composition, Body proportion, Skinfolds, Fat mass, Periodization, Playing position, Physiology, Metabolism

## Abstract

The aims of the study were: 1) to evaluate the changes in anthropometric variables, body composition, somatotype and body proportions of elite basketball players throughout the pre-season period; 2) to evaluate the changes in anthropometric variables, body composition, somatotype and body proportions of elite basketball players throughout the in-season period; and 3) to observe if the age and position influenced the variables analyzed. A total of 17 players belonging to the men’s ACB league team competing in the Euroleague (age = 23.42 ± 5.28 years-old) participated in the study. The players underwent an anthropometric measurement before and after the pre-season, as well as four evaluations throughout the in-season. Anthropometric indices, somatotype components according to Heath and Carter, and adiposity were calculated. The results show that during the pre-season, body mass, BMI, sum of 6 and 8 skinfolds, waist/hip ratio, adipose tissue (kg), adipose tissue percentage, and endomorphy decreased, while ectomorphy increased. However, no significant changes were found in the variables analyzed throughout the season; except for endomorphy, which increased along the in-season. Playing position and age did not have a significant influence on the changes in the anthropometric variables throughout the pre-season and the in-season. In conclusion, while changes in the anthropometric variables in the pre-season were observed, these remained the same during the in-season.

## Introduction

The analysis of anthropometric variables has been classically used as a method to analyze the adaptations in body composition, somatotype, and body proportion of basketball players, produced by training programs to optimize their morphological profile and performance^1–3^. Furthermore, studies point out the relationship between the morphological characteristics that are most appropriate for basketball players according to the sport’s demands, range of competition, and position of the player, with the adaptation to these characteristics being essential to optimize sports performance^4–6^.

According to the Superior Sports Council of Spain, basketball is the fifth most practiced sport in Spain, with 2,336,000 players a year, which represents 5.5% of the population that practices sports. The percentage of basketball players is higher among men and in the 15–24 age group^7^. Basketball seasons, as in other team sports, are classically divided into a preparation period (pre-season) and a competition period (in-season). The shortest phase is usually the preparation phase, lasting 5 weeks in elite teams due to the Euroleague Framework Agreement^8^. The objective of this phase is to create a good physical, tactical, technical foundation and psychological preparation for the in-season. Taking advantage of the fact that since there are no official competitions during this phase, it is possible to allocate a higher volume of load to the training sessions^9,10^. In fact, during this phase, it is very common to find up to two training sessions a day, resulting in a very high physical effort^11^.

On the contrary, during the in-season phase, which lasts 7–8 months, all the skills generated during the pre-season and throughout this period are used to achieve the maximum performance during matches^9,10^. During this period, elite players play around 60 matches, with an average of 2 or even 3 matches per week, not including daily training sessions^10,12^. Due to this high training and competition load, it is highly important to be aware of the competition calendar, phases, playoffs, and game schedules in order to plan the season properly^10,13,14^. In addition, frequent trips are made, with an impact on the quality and quantity of sleep and limited recovery time, which are stressful factors for athletes, and which condition the planning of training, rest, and recovery^10,13–16^. Therefore, training sessions during the competition calendar tend to have a lower volume and intensity than during the pre-season^13,17^.

Given that the training volume and eating habits cause the most changes in anthropometric variables, body composition, somatotype, and body proportion^18^, the variation in training loads between the pre-season and in-season phases could result in fluctuations in the composition and morphological characteristics of the basketball players^15,19^. More specifically, in team sports such as basketball, changes in body composition and energy balance have been observed in specific phases of a in-season, with players showing greater decreases in fat mass at the beginning of the pre-season^20^. However, during the in-season period, athletes tend to be in energy balance, so the changes in body composition are less significant. These data suggest that variations in composition and energy balance may be periodic at specific times in the season^18^. In addition, Hogarth et al.^21^ found changes in fat mass and lean mass in the pre-season period, but no changes were found on the same variables from the beginning to the end of the in-season period in female netball players. Similarly, in elite male football players, fat mass was found to drastically decrease in the pre-season and up to the middle of the in-season, although no significant differences were found in the second half of the season^22^.

Similarly, a significant decrease in body mass, sum of 6 and 8 skinfolds, as well as fat percentage and fat mass in the first part of the pre-season (first two weeks of training) have been found in male professional basketball players^23^. However, to the authors’ knowledge, no research has analyzed the changes in these variables in men’s elite basketball players or a population similar to the one included in the present study, although in women’s basketball, the results are contradictory. On the one hand, Ladwid et al.^24^ found no significant changes in fat percentage during the pre-season, and up to two weeks after the end of the season in female college basketball players. On the other hand, Ramírez-Bravo et al.^25^ found an increase in body mass and a decrease in body fat in the first part of the season, and the maintenance of the variables in the second part of the season.

Despite the popularity of basketball as a professional sport, there is a lack of research on the changes that occur in anthropometric variables, body composition, somatotype, and body proportion throughout the entire season (preparation [pre-season] and competition [in-season] periods) that would allow us to know if the current training loads and competitions can be used to optimize the sports performance of elite male players. Therefore, the aims of the study were: 1) to evaluate the changes in anthropometric variables, somatotype, body composition, and body proportions of elite basketball players throughout the pre-season period; and 2) to evaluate the changes in anthropometric variables, somatotype, body composition, and body proportions of elite basketball players throughout the in-season period; and 3) to observe if age and position influenced the differences in the variables analyzed.

## Methods

### Design

Retrospective longitudinal cohort study. The study design followed the guidelines of the Declaration of Helsinki and the World Medical Association codes. Approval from the ethics committee of the San Antonio Catholic University of Murcia (code CE012201) was obtained before initiating the study. In addition, participants were informed of the protocol to be performed and subsequently signed the informed consent form prior to data collection.

### Participants

The sample size calculation was performed with Rstudio software (version 3.15.0, Rstudio Inc., Boston, MA, USA). The significance level was set a priori at *α* = 0.05. The standard deviation (SD) was set according to the sum of 8 skinfolds from previous studies (SD = 1.95)^23^. With an estimated error (d) of 0.89; the sample size needed was 17 subjects.

The sample was selected by non-probabilistic, convenience sampling. In the present study, 22 volunteer elite basketball players from a men’s ACB league team classified to play in the Euroleague were part of the initial sample, with a mean age of 23.42 ± 5.28 years for two consecutive seasons. The inclusion criteria were: 1) male players belonging to the roster of an ACB league basketball team. The exclusion criteria were: 1) having suffered an injury that prevented from normal training or competition during the pre-season or in-season, 2) not having completed all the measurements in the pre-season or the in-season of at least one of the seasons, and 3) having changed their sports practice habits outside training or competition or their eating habits during the in-season.

Based on these criteria, five players were excluded (two for having suffered an injury and three for not having completed all the measurements in at least one season), with the final sample of the study being 17 players. The characteristics of the sample can be found in Table 1.

**Table 1:** Characteristics of the basketball players included.

Variable	M ± SD	Min–Max
Age (years)	23.42 ± 5.28	18.06–33.79
Years of experience in basketball	16.98 ± 6.22	9.00–24.00
Body mass (Kg)	96.91 ± 15.10	43.90–118.70
Height (cm)	196.89 ± 11.67	179.70–212.70
Arm span (cm)	200.53 ± 12.19	182.10–222.30
Adipose tissue (Kg)	27.03 ± 9.29	14.93–42.63
% Adipose tissue	27.35 ± 5.85	18.23–35–92

	*n* (%)	
Position	Point guard: *n* = 3	
	Shooting guard: *n* = 2	
	Center: *n* = 6	
	Small forward: *n* = 4	
	Power forward: *n* = 2	

The training sessions were scheduled by the coach and the team’s physical trainer, with 10 sessions per week spread over six days during the pre-season and six sessions per week spread over five days during the in-season. On their part, the players did not receive any nutritional advice from the club during the aforementioned seasons. In addition, in order to know whether the players had changed their sports practice habits outside training and matches, or whether they had changed their eating habits, the following questions were asked in each measurement session after the first one^26^: 1) physical exercise habits: “Have you done other physical exercise—such as running, going to the gym, swimming, walking at a pace that does not allow you to talk easily, etc.—simultaneous to the basketball training and competition? Which one? How long ago? How many days and hours per week?”; and 2) dietary habits: “Have you been on a diet since the last evaluation or are you on a diet now?” and “Have you significantly changed your dietary habits from the last evaluation (for example, do you eat more/less fruit and vegetables, nuts, meat, fish, cereals, pasta, fast food, etc.? How? When? Why?)”.

### Measurements

#### Kinanthropometric assessment

The participants underwent kinanthropometric measurements by a level 4 anthropometrist according to the criteria established by the International Society for the Advancement of Kynanthropometry (ISAK)^27^. Seventeen measurements were evaluated: three basic measurements (body mass, height, and arm span), eight skinfolds (triceps, subscapular, biceps, iliac crest, supraspinal, abdominal, thigh, and calf), five girths (relaxed arm, flexed and contracted arm, waist, hips, and calf) and three breaths (humerus, bistyloid and femur). Each variable was evaluated a minimum of two times, with the mean between both measurements used as the final value. If the difference between these measurements was greater than 5% for skinfolds or 1% for the rest of the measurements, a third measurement was performed, taking the median as the definitive value.

With these data, the following were calculated: body mass index (BMI)^28^, corrected arm and calf girth (corrected girth = girth–π x skinfold)^28^, sum of six (triceps, subscapular, subscapular, supraspinal, abdominal, thigh and calf) and eight skinfolds (triceps, subscapular, biceps, iliac crest, supraspinal, abdominal, thigh, and calf)^28^, waist/hip ratio (waist girth/hips girth)^28^, adipose tissue in kg and percentage^29^, cross-sectional muscle area of the arm and calf (cross-sectional muscle area = [girth − skinfold × π^2/4 π])^28^, and the somatotype, with its components of endomorphy, mesomorphy and ectomorphy with the method by Heath-Carter^30^.

The anthropometric assessment was performed on all players during the pre-season and during the playing (competitive) in-season. All the measurements were taken on the same day of the week (Monday) and by the same trained researchers. For the pre-season assessment, a first measurement was taken on the first day of training (Pre-season). The second measurement was taken the last week of the pre-season, five weeks after, with this week also being the first week of the in-season. For the evaluation of the in-season, the post–pre-season measurement was taken as the first measurement of the in-season (In-season 1), after which a measurement was taken every six to eight weeks until the end of the season (In-season 2 to In-season 4 measurements).

Measurements were taken at a temperature of 25 °C, between 12:00 and 14:00 in the afternoon. The players had not performed any previous physical exercise that day, nor vigorous exercise 18 h before. As for food, no heavy meals were ingested 24 h before the data collection on the dates established for taking the measurements.

The instrument used for taking basic measurements included a SECA 213 stadiometer (Seca, Germany) for measuring height, with an accuracy of 0.1 cm. A Seca 862 scale (SECA, Germany) with an accuracy of 100 g, was used to measure body mass. The skinfolds were measured with a Holtain caliper (Holtain Ltd., UK) with an accuracy of 0.2 mm. A Lufkin W606PM tape measure (Lufkin, USA) was used for girth measurements, and a Holtain small sliding caliper (Holtain Ltd., United Kingdom), with a 0.1 mm accuracy, was used for bone breaths. All the instruments were calibrated prior to the different measurements.

### Statistical analysis

The normality distribution of the sample was analyzed with the Shapiro–Wilk test. Skewness and kurtosis of the sample and homogeneity were checked with Levene’s test. All the variables showed a normal distribution; therefore, an analysis based on parametric tests was performed. For the descriptive analysis, the mean ± standard deviation of the different variables was calculated. To calculate the differences between pre-season and in-season measurements, a repeated measures analysis of variance (ANCOVA) was used, with the covariables analyzed being playing position and age. The significance level was set a priori at *p* < 0.05. In addition, a Bonferroni post hoc analysis was performed on the variables that had shown significant differences, adjusting the *p*-value. Effect size was calculated with partial eta squared (*η*^2^_p_), the evaluation criteria used were 0.01–0.06 for a low effect, 0.06–0.14 for a medium effect, and high if the value was 0.14 or higher^31,32^. The SPSS statistical program (version 25.0) was used for the statistical analysis.

## Results

A significant decrease in body mass, BMI, sum of 6 and 8 skinfolds, waist/hip ratio, adipose tissue in kg and percentage, as well as endomorphy was observed, with the players obtaining lower results after the pre-season in all cases; in the case of ectomorphy, an increase was observed after the pre-season (Tables 2 and 3). When considering the covariates age and position, these variables were not shown to significantly influence the model (Tables 2 and 3).

**Table 2:** Mean ± standard deviation of anthropometric variables, somatotype, adiposity and body proportions, and differences between them and as a function of playing position and age, significance value in pre- and post–pre-season measurements.

	Measurements							Measurements*Position					Measurements*Age				
Variable	Pre-season (M ± SD)	Post–pre-season (M ± SD)	*p*-value	F	95% CI min	95% CI max	ES	*p*-value	F	95% CI min	95% CI max	ES	*p*-value	F	95% CI min	95% CI max	ES
Body mass (Kg)	96.91 ± 15.10	95.70 ± 14.04	0.009*	9.07	88.55	104.07	0.38	0.502	0.48	86.10	105.16	0.04	0.704	0.15	88.57	104.05	0.01
Height (cm)	196.89 ± 11.67	196.90 ± 11.67	0.333	1.00	190.68	203.12	0.06	0.193	1.92	187.60	202.62	0.15	0.772	0.09	190.42	203.38	0.01
Corrected arm girth (cm)	30.19 ± 1.72	30.19 ± 1.61	0.991	0.00	29.32	31.07	0.00	0.296	1.20	29.31	31.43	0.10	0.983	0.00	29.62	30.78	0.00
Corrected calf girth (cm)	37.73 ± 3.05	37.82 ± 2.91	0.317	1.07	36.19	39.37	0.07	0.369	0.88	35.73	39.75	0.07	0.332	1.01	36.31	39.25	0.07
CSA arm (cm^2^)	74.78 ± 8.37	72.76 ± 7.76	0.983	0.00	68.57	76.98	0.00	0.302	1.17	68.47	78.76	0.10	0.989	0.00	69.98	75.57	0.00
CSA calf (cm^2^)	113.97 ± 18.47	114.51 ± 17.64	0.367	0.87	104.64	123.86	0.06	0.336	1.01	101.88	126.25	0.08	0.302	1.15	105.33	123.17	0.08
BMI (Kg/cm^2^)	25.85 ± 1.88	24.56 ± 1.74	0.013*	7.87	23.75	25.68	0.34	0.564	0.35	23.83	26.12	0.03	0.536	0.40	24.03	25.40	0.03
Sum 6 SF (mm)	76.12 ± 27.36	67.52 ± 21.62	0.001*	15.99	58.89	84.76	0.52	0.471	0.56	55.16	82.69	0.05	0.481	0.53	58.38	85.27	0.04
Sum 8 SF (mm)	98.30 ± 34.79	85.73 ± 26.94	0.001*	17.88	75.74	108.30	0.54	0.353	0.94	71.13	106.39	0.08	0.534	0.41	75.09	108.95	0.03
Waist/hip ratio	0.83 ± 0.03	0.82 ± 0.02	0.001*	18.28	0.81	0.84	0.55	0.097	3.29	0.81	0.85	0.23	0.340	0.98	0.81	0.84	0.07
Adipose tissue (kg)	27.03 ± 9.29	25.00 ± 7.86	0.002*	14.69	21.46	30.57	0.50	0.422	0.69	19.87	29.69	0.06	0.719	0.14	21.30	30.73	0.01
% Adipose tissue	27.35 ± 5.85	25.73 ± 5.02	0.001*	16.54	23.67	29.42	0.52	0.498	0.49	22.54	28.43	0.04	0.482	0.52	23.68	29.40	0.04
Endomorphy	2.84 ± 0.95	2.51 ± 0.70	0.006*	10.08	2.25	3.12	0.40	0.698	0.16	2.10	3.10	0.01	0.732	0.12	2.23	3.13	0.01
Mesomorphy	4.72 ± 0.91	4.72 ± 0.91	0.988	0.00	4.24	5.21	0.00	0.803	0.07	4.46	5.48	0.01	0.739	0.12	4.31	5.15	0.01
Ectomorphy	2.88 ± 0.83	2.99 ± 0.86	0.016*	7.30	2.49	3.39	0.33	0.639	0.23	2.23	3.24	0.02	0.610	0.27	2.59	3.30	0.02

CSA: Cross-sectional area of muscle; BMI: Body Mass Index; SF: Skinfolds; *p<0.05.

**Table 3:** Pairwise comparison and statistical results of anthropometric variables, somatotype, adiposity and body proportions that showed significant change during the pre-season (pre- vs. post–pre-season measurements).

Variable	Diff. measurements	*p*-value	95% CI min	95% CI max
Body mass (Kg)	1.22 ± 0.41	0.009	0.36	2.08
BMI (Kg/cm^2^)	0.29 ± 0.10	0.013	0.07	0.50
Sum 6 SF (mm)	8.60 ± 2.15	0.001	4.02	13.18
Sum 8 SF (mm)	12.56 ± 2.97	0.001	6.23	18.90
Waist/hip ratio	0.01 ± 0.00	0.001	0.01	0.02
Adipose tissue (kg)	2.03 ± 0.53	0.002	0.90	3.15
% Adipose tissue	1.62 ± 0.40	0.001	0.77	2.47
Endomorphy	0.34 ± 0.11	0.006	0.11	0.56
Ectomorphy	−0.12 ± 0.04	0.02	−0.21	−0.03

CSA: Cross-sectional area of muscle; BMI: Body Mass Index; SF: Skinfolds.

No significant changes were shown in any of the variables during the in-season, except for endomorphy, which increased at the end of the in-season (Table 4). When the pairwise comparison was performed, no differences were found between in-season measurements, so these data have not been included in any table. Position and age did not have a significant influence on the model either (Table 4).

**Table 4:** Mean ± standard deviation of anthropometric variables, somatotype, adiposity and body proportions, and differences between them and as a function of playing position and age, significance values in measurements during the in-season.

	Measurements									Measurements*Position					Measurements*Age				
Variable	In-season 1 (M ± SD)	In-season 2 (M ± SD)	In-season 3 (M ± SD)	In-season 4 (M ± SD)	*p*-value	F	95% CI min	95% CI max	ES	*p*-value	F	95% CI min	95% CI max	ES	*p*-value	F	95% CI min	95% CI max	ES
Body mass (Kg)	95.70 ± 14.04	95.02 ± 14.32	95.60 ± 13.80	95.86 ± 14.16	0.863	0.03	83.73	100.39	0.00	0.663	0.20	86.13	97.99	0.02	0.808	0.07	81.25	106.73	0.01
Height (cm)	196.90 ± 11.67	195.80 ± 13.63	195.80 ± 13.63	195.80 ± 13.63	1.000	0.00	188.52	203.17	0.00	1.000	0.00	192.85	198.84	1.00	1.000	0.00	184.32	206.45	0.00
Corrected arm girth (cm)	30.19 ± 1.61	30.17 ± 1.25	30.65 ± 1.46	30.51 ± 1.48	0.544	0.40	28.90	30.78	0.04	0.633	0.24	28.84	30.84	0.03	0.336	1.09	29.34	30.72	0.15
Corrected calf girth (cm)	37.82 ± 2.91	37.48 ± 2.83	37.61 ± 2.80	37.49 ± 2.68	0.775	0.09	35.39	39.03	0.01	0.752	0.11	35.49	38.93	0.01	0.111	3.49	34.97	40.13	0.37
CSA arm (cm^2^)	72.76 ± 7.76	72.55 ± 5.89	74.91 ± 7.06	74.25 ± 7.09	0.525	0.44	66.60	75.42	0.04	0.634	0.24	66.33	75.68	0.03	0.292	1.33	68.63	75.11	0.18
CSA calf (cm^2^)	114.51 ± 17.64	112.33 ± 16.88	113.09 ± 16.64	112.36 ± 15.99	0.827	0.05	99.79	121.65	0.01	0.808	0.06	100.32	121.12	0.01	0.110	3.50	97.16	128.41	0.37
BMI (Kg/cm^2^)	24.56 ± 1.74	24.68 ± 1.63	24.85 ± 1.58	24.90 ± 1.49	0.857	0.03	22.84	25.01	0.00	0.698	0.16	22.76	25.08	0.02	0.747	0.11	23.49	25.52	0.02
Sum 6 SF (mm)	67.52 ± 21.62	69.90 ± 21.95	69.28 ± 17.71	71.80 ± 18.60	0.773	0.09	48.29	74.00	0.01	0.844	0.04	49.42	72.87	0.01	0.492	0.54	44.41	83.74	0.08
Sum 8 SF (mm)	85.73 ± 26.94	88.10 ± 26.14	89.26 ± 22.21	92.68 ± 23.68	0.899	0.02	61.94	94.08	0.00	0.743	0.11	63.56	92.47	0.01	0.497	0.52	57.27	106.54	0.08
Waist/hip ratio	0.82 ± 0.02	0.83 ± 0.02	0.83 ± 0.02	0.84 ± 0.01	0.549	0.38	0.81	0.84	0.04	0.392	0.81	0.81	0.84	0.08	0.342	1.07	0.81	0.85	0.15
Adipose tissue (kg)	25.00 ± 7.86	25.35 ± 9.05	25.15 ± 8.09	25.73 ± 8.32	0.778	0.08	18.44	28.01	0.01	0.857	0.03	19.74	26.71	0.00	0.534	0.43	16.42	31.30	0.07
% Adipose tissue	25.73 ± 5.02	26.16 ± 5.56	25.88 ± 4.91	26.37 ± 4.87	0.644	0.23	21.74	27.92	0.02	0.841	0.04	22.28	27.38	0.01	0.357	1.00	20.44	29.29	0.14
Endomorphy	2.51 ± 0.70	2.58 ± 0.60	2.52 ± 0.42	2.72 ± 0.59	0.009	0.92	1.88	2.70	0.00	0.563	0.36	1.89	2.69	0.04	0.863	0.03	1.75	3.01	0.01
Mesomorphy	4.72 ± 0.91	4.78 ± 1.04	4.82 ± 1.02	4.81 ± 1.02	0.519	0.45	3.91	5.18	0.04	0.465	0.58	4.07	5.03	0.06	0.780	0.09	4.06	5.44	0.01
Ectomorphy	2.99 ± 0.86	2.88 ± 0.97	2.80 ± 0.99	2.78 ± 0.92	0.887	0.02	2.63	3.82	0.00	0.674	0.19	2.73	3.71	0.02	0.713	0.15	2.38	3.49	0.02

CSA: Cross-sectional area of muscle; BMI: Body Mass Index; SF: Skinfolds.

## Discussion

The main objective of the study was to analyze the changes observed in anthropometric variables, body composition, somatotype, and body proportions of male professional basketball athletes in the pre-season. A significant decrease was found in all parameters related to adiposity, i.e., adipose tissue in kg and percentage, sum of 6 and 8 skinfolds, as well as in endomorphy. The results coincide with those found in previous studies conducted in men’s basketball players^20,23^, where a significant decrease in fat, in absolute value and percentage, sum of skinfolds, and the endomorphic component of the somatotype, were found in the pre-season The training load tends to be higher during the pre-season period^18,21^, where sessions are characterized by more resistance work and a high frequency of weekly training sessions and friendly matches^33–35^. So, changes in adipose variables could be related to energy expenditure because of the training volume during the pre-season^24^.

Another outstanding result of the study was the decrease in waist/hip ratio found in professional basketball players in the pre-season. This variable has been analyzed in longitudinal studies carried out with elite team sport athletes, and coinciding with the present study, a significant decrease was found both in the pre-season and in the in-season^36^. These changes could be due to the fact that the high training load in the pre-season could decrease not only adipose tissue in general, but specifically in the abdominal area^36^. However, up to date, no previous studies have analyzed this issue in elite basketball players. Therefore, the results of this investigation need to be tested in future studies.

Another result was a decrease in BMI in the pre-season, which is influenced by changes in body mass and height. Considering that the body height of the players did not change during this period, as this population has already stopped growing^37^, the changes in this parameter would be conditioned by changes in body mass. In this regard, it must be taken into consideration that body mass alone is not able to differentiate between the fat and lean components^38^. Therefore, the reason for the change found in body mass could be the decrease in the adipose component observed in the study. The results are similar to those observed in professional football players, where a significant decrease in body mass was observed during the pre-season^35^. However, other studies have not found a significant change in this variable during the pre-season in female basketball players, which may be due to their increase in muscle mass^39^.

Regarding the variables related to muscle development, no changes were found during the pre-season, neither in their general evaluation, nor after the individual analysis of their playing positions. No previous study has examined this issue in basketball players. However, similar results have been found in other team sports, such as professional football players^34^. Although an increase in muscle mass related to training would be expected, it is important to note that these changes are noticeable mainly in untrained individuals, who are subjected to a new stimulus; as the individual has more experience in training with adaptations, a higher intensity of effort may be necessary to observe minimal changes^24^. In this sense, the absence of muscle mass gain in the pre-season could be related to the high volume of training during the pre-season (10–12 sessions per week) and the predominance of endurance sessions^18,40^, which could result in a caloric deficit that would not have enhanced muscle mass gain^34^, suggesting a relationship between changes in anthropometric variables, body composition, somatotype and body proportions, energy balance, and the specific time in the season at which they occur^18,22^. However, other studies have found an increase in muscle mass during the pre-season in team sports^21,39,40^ or even its decrease^36^. Therefore, this issue should be further analyzed in different sports, levels of competition, and sex, in future research studies.

The second objective of the research was to analyze the changes observed in the anthropometric variables, body composition, somatotype and body proportions, throughout the in-season. A curious finding of the present research was that, while the adipose tissue and the sum of skinfolds increased, with no significant differences, endomorphy increased significantly along the in-season, although in the pairwise comparison none of the pairs was significant. The fact that significant increase was found in the endomorphy, but not in the other variables related to adiposity, could be because in order to calculate endomorphy, adiposity is relativized in relation to height, which makes this variable more sensitive to changes in the skinfolds assessed for populations with higher heights^28^, such as the one in the present study.

The results coincide with those found in other studies on college basketball players ^41^ and on elite junior male basketball players^20^, where no significant decrease in majority of adiposity variables were found throughout the in-season. The absence of changes during the in-season in the variables related to adiposity, and even the increase seen in the endomorphy, could be due to the fact that once the pre-season is over, the duration and volume of the sessions decrease, as well as the frequency of resistance sessions^35^, as the training sessions are mostly devoted more to technical work, with little emphasis on conditioning^16,18,39^, and priority is also given to recovery and preparation for the match^18,40^. However, the lack of longitudinal studies analyzing the evolution of somatotype along the in-season means that this result needs to be tested in future research.

On the other hand, as observed during the pre-season, there was no significant change in variables related to muscle mass, which is in line with previous research conducted in general athletes^41^ and college basketball players^39^. This could be due to the fact that although the volume of training is lower than in the pre-season, which could result in players no longer being in energy balance for the most part^18,40^, there is also a decrease in the number of strength training sessions, limited to one per week in most cases, which could be an insufficient stimulus to provoke changes in this variable in elite athletes^35^.

Differences in body mass were not observed neither, which is in line with the findings from previous studies that focused on elite basketball male junior players^20,41^. These results could be conditioned by the absence of changes in the adipose and muscular components.

Based on the results of the study, it would be necessary to carry out individual training and diet interventions in athletes whose morphology and composition is not adequate at the end of the pre-season period, as training with the group may not have had a sufficiently significant effect^21,24,35^. This could be because the demands of the competitive period in terms of game and training schedules, including frequent travel, which interrupts their usual routine, could limit recovery, resulting in physical and psychological stress in the athlete. Therefore, to compensate for this, it is necessary to decrease the volume of training at this stage of the in-season^14,16^. However, further studies are needed to analyze the influence of other factors such as experience and biological age^21,40^ on the changes observed in these factors.

A final objective of the research was to observe whether the covariates of age and position had an influence on the changes observed in the anthropometric variables, body composition, somatotype and body proportions. In this sense, although the position of a player within a team may influence the changes in anthropometric variables related to the various workloads^22^, in the present study, the position did not have an influence on the changes in the variables, neither in the pre-season nor during the in-season. In the absence of previous research on this issue in basketball, it should be noted that the results of the research are in agreement with those found in American football athletes^36^ and rugby players^40^, with similar changes observed in players, depending on their playing position throughout the season. However, in the case of rugby players, the change was slightly greater in forwards than in backs, which could be due to the different physiological demands of this sport depending on the playing position^40^.

In the study, age was also not found to have a significant influence on the changes in anthropometric variables, body composition, somatotype, and body proportions. Previous cross-sectional studies have shown that anthropometric variables, body composition, somatotype and/or body proportion could show differences depends on age in basketball players^20^. Nevertheless, no previous longitudinal studies have analyzed whether changes in morphological variables along the pre-season or in-season differ according to age in basketball players. However, when comparing the results of the study in this area with those previously carried out in other team sports, our findings are in agreement with previous studies conducted in Australian senior professional football players, in which it was found that the changes in these variables were similar throughout a season, with no influence of age, although differences were observed in the anthropometric variables, body composition, somatotype and body proportions as a function of age^42^.

The study is not free of limitations. The main limitation is the size of the sample and the absence of a control group, as well as the evaluation of only one elite team, which limits the possibility of generalizing the results to professional basketball athletes as a population. In addition, there was no control in terms of individual training load, and the lack of a nutritional evaluation must be considered, despite the monitorization performed to detect changes in this variable. Future lines of research could consider the evaluation of each stage of an elite basketball season, though the analysis of individual training loads and nutrition, which would facilitate understanding the trends observed in the changes in anthropometric variables, body composition, somatotype and body proportions over time.

## Conclusion

Elite basketball players showed a decrease in adiposity variables and body mass throughout the pre-season, without significant changes observed in variables related to muscle development. During the in-season, no significant changes were found in the anthropometric variables, body composition, somatotype and body proportions, with the exception of the endomorphic component of the somatotype, which showed a significant increase over the in-season. Furthermore, neither position nor age had a significant influence on the changes of the variables analyzed. As a result of the findings, the professionals involved in the physical preparation and nutrition of elite basketball teams could define plans based on the specific demands and objectives of each player, with individualized training and nutrition interventions being necessary in the event that an optimal morphology and body composition have not been achieved after the pre-season.

## Data Availability

The datasets used and/or analyzed during the current study are available from the corresponding author on reasonable request.
